# Analysis of the learning curve for neuroendoscopic evacuation of basal ganglia hemorrhage

**DOI:** 10.3389/fsurg.2026.1753445

**Published:** 2026-06-23

**Authors:** Xuqiang Wang, Fei Gao, Qiang Zhu, Yunfeng Jia, Ganggang She

**Affiliations:** Department of Neurosurgery, Yan'an University Affiliated Hospital, Yan’an, China

**Keywords:** hypertensive intracerebral hemorrhage, intracerebral hematoma evacuation, learning curve, minimally invasive treatment, neuroendoscopy

## Abstract

**Background:**

Neuroendoscopic evacuation has become an important minimally invasive approach for the treatment of basal ganglia hemorrhage. However, its technical complexity requires a well-defined learning curve, and evidence regarding learning curve characteristics, particularly among experienced surgeons, remains limited.

**Objective:**

To evaluate the learning curve of neuroendoscopic evacuation for basal ganglia hemorrhage and to determine the number of cases required to achieve technical proficiency.

**Methods:**

A retrospective analysis was conducted on 120 consecutive patients with basal ganglia hemorrhage who underwent neuroendoscopic evacuation performed by a single experienced neurosurgeon. The learning curve was assessed using cumulative sum (CUSUM) analysis based on operative time. A segmented regression model and a power-law model were applied to characterize the learning curve. Surgical outcomes, hemostasis strategies, and postoperative imaging indicators were compared across different learning phases.

**Results:**

The CUSUM analysis identified a clear learning inflection point at approximately the 25th case. Operative time decreased significantly across phases (1.77 ± 0.47 h vs. 1.28 ± 0.40 h vs. 1.23 ± 0.42 h, *P* < 0.01), while the hematoma evacuation rate increased significantly (84.90% ± 3.59% vs. 93.27% ± 3.65% vs. 92.92% ± 3.41%, *P* < 0.01). Most surgical performance indicators improved significantly between the initial and proficiency phases, with no significant differences observed between the later phases, indicating performance stabilization. In addition, the use of electrocoagulation and hemostatic materials decreased significantly, accompanied by improvements in postoperative imaging indicators and a reduction in overall complication rates (*P* = 0.005).

**Conclusion:**

Significant differences exist in neuroendoscopic evacuation of basal ganglia hematomas across different learning phases. These findings can provide guiding value for clinicians to improve surgical efficiency and quality.

## 1 Introduction

Hypertensive intracerebral hemorrhage is a hemorrhagic cerebrovascular disease caused by the rupture of intracerebral blood vessels due to long-term hypertension, which is associated with high morbidity and mortality (1, 2). Hematoma evacuation strategies focusing on maximizing neurofunctional protection have become the primary approach for the surgical management of hypertensive intracerebral hemorrhage (3, 4). Neuroendoscopy has emerged as a major minimally invasive treatment for hypertensive intracerebral hemorrhage, boasting advantages such as minimal trauma, high hematoma evacuation rate, and rapid postoperative recovery (5–7). However, this technique is technically demanding and requires substantial learning and practice to master. This study focused exclusively on hypertensive intracerebral hemorrhage located in the basal ganglia, aiming to summarize and analyze the key technical priorities of neuroendoscopic intracerebral hematoma evacuation at different learning phases, thereby providing a reference for improving clinical treatment efficiency and implementing standardized operational procedures.

However, there remains a lack of systematic evidence regarding procedural optimization and learning curve characteristics in neuroendoscopic evacuation of basal ganglia hematoma, particularly in experienced surgical settings. Most existing studies focus on novice surgeons, while data derived from experienced neurosurgeons remain limited. Therefore, this study aimed to evaluate the learning curve of neuroendoscopic hematoma evacuation and to provide practical insights for surgical training and standardization.

## 2 Materials and methods

### 2.1 Study design and patient selection

This retrospective single-center observational study included consecutive patients who underwent neuroendoscopic evacuation of basal ganglia hematoma at the Department of Neurosurgery, Affiliated Hospital of Yan'an University, between September 2021 and June 2023.

This study was conducted and reported in accordance with the STROBE (Strengthening the Reporting of Observational Studies in Epidemiology) guidelines for observational studies.

All procedures were performed by a single senior neurosurgeon experienced in neuroendoscopic techniques. The 120 consecutive cases were chronologically divided into three sequential learning phases (40 cases per phase): initial exploration phase, technical proficiency phase, and stable optimization phase. This predefined grouping strategy was intended to allow balanced comparison across different stages of the surgical learning curve.

The inclusion criteria (8, 9) were as follows: (1) age 18–80 years; (2) confirmed diagnosis of hypertensive intracerebral hemorrhage; (3) hematoma located in the unilateral basal ganglia (putamen, caudate nucleus, or external capsule); (4) time from onset to surgery ≤72 h; (5) meeting surgical indications; (6) first episode of intracerebral hemorrhage without prior intracranial surgery.

The exclusion criteria were: (1) secondary causes of hemorrhage, including coagulopathy, anticoagulant/antiplatelet use, vascular malformations, aneurysms, tumors, or trauma; (2) hematomas involving the thalamus, brainstem, cerebellum, or extensive intraventricular hemorrhage; (3) severe systemic diseases (e.g., cardiac, pulmonary, hepatic, or renal failure); (4) preoperative cerebral herniation; (5) contraindications to surgery (e.g., infection, coagulation abnormalities with INR >1.4 or platelets <50  ×  10⁹/L); (6) simultaneous treatment of other intracranial lesions; (7) pre-existing severe neurological disorders; and (8) incomplete key perioperative clinical or imaging datasets or loss to follow-up.

The study protocol was approved by the Institutional Review Board of the Affiliated Hospital of Yan'an University. The requirement for individual informed consent was waived owing to the retrospective nature of the study.

### 2.2 Surgical procedure

All procedures were performed under general anesthesia by the same experienced neurosurgical team. Patients were positioned supine with the head elevated approximately 15°–30° and rotated slightly contralateral to the lesion side to optimize the surgical trajectory and facilitate gravitational brain relaxation.

Preoperative computed tomography (CT) imaging was carefully reviewed to determine the optimal cortical entry point and surgical trajectory. Surgical trajectory planning was primarily based on hematoma location, maximal long-axis orientation of the hematoma cavity, ventricular extension, and the principle of minimizing cortical and white matter injury. In selected anatomically complex cases, neuronavigation assistance was additionally utilized to optimize puncture trajectory accuracy.

A frontal transcranial approach was routinely adopted. A linear scalp incision (∼6 cm) was made approximately 9 cm superior to the glabella and 3–3.5 cm lateral to the midline on the affected side. After creation of a small craniotomy (approximately 2–3 cm in diameter), the dura mater was opened in a cruciate fashion.

Following careful corticotomy through a relatively avascular cortical region, a puncture needle or drainage catheter was advanced toward the hematoma cavity under anatomical guidance to achieve preliminary decompression and confirm trajectory orientation. Sequential dilation of the working corridor was then performed, and a transparent working sheath was introduced into the hematoma cavity.

Under rigid neuroendoscopic visualization, hematoma evacuation was performed in a controlled distal-to-proximal manner while preserving surrounding neural structures. Intraoperative decompression was achieved gradually to minimize abrupt intracranial pressure shifts and reduce the risk of rebleeding. Hemostasis was carefully achieved using bipolar coagulation, monopolar coagulation when necessary, continuous irrigation, and absorbable hemostatic materials.

For patients with intraventricular hemorrhage (IVH) and ventricular enlargement, external ventricular drainage (EVD) was additionally performed according to ventricular involvement severity, cerebrospinal fluid circulation status, and perioperative intracranial pressure management requirements.

At the end of the procedure, a drainage catheter was placed within the residual hematoma cavity or ventricular system when indicated, followed by standard layered wound closure.

Several technical aspects of the procedure gradually evolved during the learning curve. With increasing surgical experience, improvements were observed in trajectory selection accuracy, endoscopic spatial orientation, hematoma cavity identification, sheath positioning stability, intraoperative hemostatic efficiency, and coordination between suction and decompression techniques. In addition, perioperative workflow standardization and cerebrospinal fluid drainage management were progressively optimized during later surgical phases.

### 2.3 Outcome measures and data collection

Clinical, imaging, operative, and perioperative management data were retrospectively collected from electronic medical records, operative reports, and radiological databases.

Baseline variables included sex, age, diabetes status, smoking and alcohol history, and preoperative blood pressure control. Hematoma-related variables included hematoma volume (calculated using 3D Slicer), morphology (regular vs. irregular), intraventricular hemorrhage, external ventricular drainage, perilesional edema volume, and hematoma CT value. The time from onset to surgery was recorded as a preoperative temporal variable. Surgical and postoperative parameters included operative time (from skin incision to closure), use of electrocoagulation and hemostatic materials, hematoma evacuation rate, drainage tube removal time, postoperative changes in abnormal density volume, and volume of air-like low-density areas. Clinical outcomes included rebleeding, intracranial infection, and non-operative site hematoma. The hematoma evacuation rate was calculated as: (preoperative volume−residual volume)/preoperative volume  ×   100%.

### 2.4 Statistical analysis

All statistical analyses were performed using SPSS software (IBM Corp., Armonk, NY, USA). Continuous variables were expressed as mean ± standard deviation (SD). Data normality was assessed using the Shapiro–Wilk test, and homogeneity of variance was evaluated using Levene's test.

For comparisons between the three learning phases, one-way analysis of variance (ANOVA) was used for normally distributed data with equal variances, followed by Bonferroni *post hoc* tests. Non-normally distributed data were analyzed using the Kruskal–Wallis test. Categorical variables were summarized as frequencies and percentages, and compared using the chi-square test or Fisher's exact test, as applicable.

Learning curve analysis was performed using cumulative sum (CUSUM) analysis based on operative time. The CUSUM value for the *n*-*th* case was calculated as:

$$CUSUM_{n}=\overset{n}{\underset{i=1}{\sum }}(X_{i}-\mu )$$

where *Xi* represents the operative time for the *i-th* procedure and *μ* denotes the overall mean operative time of all procedures.

To further characterize the learning curve, a segmented regression model was applied based on the identified inflection point. A power-law model was used to fit the initial learning phase:

$$T(n)=a\cdot n^{-b}$$

where *T*(*n*) represents the operative time for the *n*-*th* case, *a* is a scaling constant, and *b* reflects the learning rate (larger *b* values indicate faster skill acquisition). After the inflection point, linear regression analysis was used to characterize performance stabilization.

Model fitting was evaluated using the coefficient of determination (*R*^2^). All statistical tests were two-sided, and a *P*-value < 0.05 was considered statistically significant.

Only patients with complete datasets relevant to the corresponding analyses were included in the final statistical evaluation to minimize information bias and maintain data consistency.

## 3 Results

### 3.1 Baseline characteristics

There were no significant differences in baseline characteristics among the initial exploration, technical proficiency, and stable optimization phases (all *P* > 0.05), indicating good comparability between groups (*P* > 0.05, Table 1).

**Table 1:** Comparison of baseline patient characteristics across learning phases.

Characteristic	Initial exploration (*n* = 40)	Technical proficiency (*n* = 40)	Stable optimization (*n* = 40)	*χ*^2^/F	*P*
Sex (Male/Female)	26/14	27/13	26/14	0.074	0.964
Diabetes (Yes/No)	11/29	12/28	12/28	0.081	0.960
Smoking/Alcohol (Yes/No)	24/16	21/19	19/21	1.272	0.529
Preop BP controlled (Yes/No)	32/8	30/10	31/9	0.287	0.866
Hematoma Morphology (Reg/Irr)	31/9	33/7	33/7	0.430	0.806
IVH (Yes/No)	7/33	10/30	9/31	0.687	0.709
EVD (Yes/No)	4/36	5/35	7/33	1.01	0.604
Age (years)	63.82 ± 9.02	65.35 ± 8.04	64.37 ± 7.96	0.341	0.711
Hematoma volume (ml)	55.45 ± 10.14	58.80 ± 8.87	58.72 ± 9.56	1.607	0.205
Edema volume (ml)	20.62 ± 6.50	23.77 ± 7.53	23.12 ± 7.47	2.141	0.122
Hematoma CT value (HU)	71.47 ± 9.08	70.45 ± 9.36	72.40 ± 9.05	0.452	0.637
Onset-to-surgery time (h)	7.72 ± 2.16	7.42 ± 1.89	7.02 ± 2.13	1.157	0.318

IVH, intraventricular hemorrhage; EVD, external ventricular drainage; Reg/Irr, regular/irregular; BP, blood pressure; Preop, preoperative.

### 3.2 Surgical outcomes across learning phases

Significant differences were observed among the three phases in operative time, hematoma evacuation rate, drainage tube removal time, postoperative abnormal density volume difference, and air-like density volume (all *P* < 0.05).

Operative time decreased significantly from 1.77 ± 0.47 h in the initial phase to 1.28 ± 0.40 h and 1.23 ± 0.42 h in the proficiency and optimization phases, respectively (*P* < 0.01). The hematoma evacuation rate increased from 84.90% ± 3.59% to 93.27% ± 3.65% and 92.92% ± 3.41% (*P* < 0.01). The time to drainage tube removal was also significantly reduced (25.27 ± 7.51 h vs. 21.82 ± 4.78 h vs. 20.32 ± 5.47 h, *P* = 0.001).

Postoperative abnormal density volume difference decreased progressively across phases (26.72 ± 5.37 mL vs. 19.05 ± 5.53 mL vs. 11.97 ± 3.00 mL, *P* < 0.01), as did the volume of air-like density areas (19.05 ± 10.70 mL vs. 10.32 ± 3.80 mL vs. 8.22 ± 2.46 mL, *P* < 0.01).

Post hoc analysis showed that all indicators differed significantly between the initial and proficiency phases (all *P* < 0.05), whereas no significant differences were observed between the proficiency and optimization phases in operative time, hematoma evacuation rate, drainage tube removal time, and air-like density volume (all *P* > 0.05), indicating stabilization of surgical performance. However, the postoperative abnormal density volume difference continued to improve significantly (*P* < 0.01) (Table 2).

**Table 2:** Comparison of surgical-related indicators across learning phases.

Surgical indicator	Initial exploration (*n* = 40)	Technical proficiency (*n* = 40)	Stable optimization (*n* = 40)	F	*P*
Operation time (hours)	1.77 ± 0.47	1.28 ± 0.4	1.23 ± 0.42	18.16	< 0.01
Hematoma evacuation rate (%)	84.90 ± 3.59	93.27 ± 3.65	92.92 ± 3.41	71.05	< 0.01
Drainage tube removal time (hours)	25.27 ± 7.51	21.82 ± 4.78	20.32 ± 5.47	7.07	0.001
Postop abnormal density vol. diff (mL)	26.72 ± 5.37	19.05 ± 5.53	11.97 ± 3.00	95.28	< 0.01
Air-like density volume (mL)	19.05 ± 10.70	10.32 ± 3.8	8.22 ± 2.46	29.25	< 0.01

### 3.3 Hemostasis strategy and complications

Significant differences were observed among the three phases in the use of electrocoagulation and hemostatic materials (both *P* < 0.01). The use of electrocoagulation decreased progressively (50.0% vs. 27.5% vs. 10.0%), as did the use of hemostatic materials (82.5% vs. 35.0% vs. 35.0%).

There were no significant differences in the incidences of rebleeding, intracranial infection, or non-operative site hematoma among the three phases (all *P* > 0.05). However, the overall complication rate decreased significantly (45.0% vs. 20.0% vs. 15.0%, *P* = 0.005) (Table 3).

**Table 3:** Comparison of hemostatic techniques and complication indicators.

Indicator	Initial exploration (*n* = 40)	Technical proficiency (*n* = 40)	Stable optimization (*n* = 40)	χ^2^	*P*
Electrocautery (Yes/No)	20/20	11/29	4/36	15.57	<0.01
Hemostatic Materials (Y/N)	33/7	14/26	14/26	24.07	<0.01
Rebleeding (Yes/No)	6/34	3/37	2/38	2.620	0.270
Intracranial Infection (Y/N)	5/35	2/38	2/38	2.160	0.339
Non-op Hematoma (Yes/No)	7/33	3/37	2/38	3.889	0.143
Total Complications (Yes/No)	18/22	8/32	6/34	10.56	0.005

Total complications: Presence of at least one of reelectrocautery bleeding, intracranial infection, or non-operative hematoma.

### 3.4 Learning curve analysis

The CUSUM analysis identified a clear inflection point at approximately the 25th case. A segmented regression model demonstrated that operative time decreased rapidly during the initial phase and gradually stabilized thereafter. The power-law model fitted the early learning phase with a coefficient of determination (R^2^ = 0.65), while a linear model provided a better fit for the later phase (R^2^ = 0.85). The overall model showed a good fit (R^2^ = 0.68).

## 4 Discussion

Neurofunctional recovery after hypertensive intracerebral hemorrhage is influenced by multiple factors, including hematoma characteristics, surgical strategy, and perioperative management. In recent years, minimally invasive approaches based on white matter tract preservation have become the preferred treatment paradigm (10, 11). Within this context, understanding the learning curve of neuroendoscopic hematoma evacuation is essential for optimizing surgical performance and improving clinical outcomes. This study specifically focused on basal ganglia hemorrhage, the most common subtype, to characterize both technical proficiency and the evolution of surgical strategies.

### 4.1 Learning curve and surgical efficiency

Previous studies on neuroendoscopic learning curves have primarily focused on novice surgeons, often reporting prolonged learning phases and greater variability in outcomes. In contrast, the present study evaluates an experienced neurosurgeon and demonstrates a relatively rapid transition to technical proficiency. This difference highlights the impact of baseline surgical experience on the learning curve. The division into three equal phases was intended to ensure a balanced comparison across different stages of the learning curve, although alternative grouping strategies based on statistical inflection points may also be considered.

This study demonstrated a clear reduction in operative time across learning phases, reflecting progressive skill acquisition. In the initial phase, the main challenges were related to spatial orientation under endoscopic visualization and accurate targeting of the hematoma cavity, often resulting in repeated adjustments and reduced efficiency. With increasing experience, optimization of surgical trajectory and bone window positioning significantly improved procedural fluency.

### 4.2 Evolution of hemostasis strategies

The advancement of hemostasis techniques represents a key component of the learning curve. In the initial phase, surgeons tended to rely excessively on Electrocautery and hemostatic materials due to limited experience in assessing perforating vessel injury and bleeding characteristics. Although effective for bleeding control (12–14), overuse of these methods may contribute to the formation of air-like density areas, primarily caused by the accumulation and expansion of hemostatic materials within the operative field.

These materials, including oxidized regenerated cellulose and gelatin-based agents, can exert mass effect and hinder brain tissue re-expansion, potentially compromising neurofunctional recovery. In addition, residual intracavitary gas may also contribute to air-like density areas, although these typically resolve over time depending on the residual volume.

With increasing experience, surgeons develop a more refined understanding of bleeding patterns and adopt a more targeted hemostasis strategy, reducing unnecessary use of adjunctive materials and improving operative field control. This transition reflects a shift from excessive to precision-based hemostasis and is associated with improved postoperative imaging outcomes.

### 4.3 Imaging outcomes and technical refinement

Postoperative imaging findings further supported the progressive refinement of surgical technique. The reduction in abnormal density volume suggests decreased surgical trauma and improved preservation of surrounding brain tissue. These improvements are likely related to enhanced instrument stability, better control of suction pressure, and adherence to refined operative principles (15, 16). In addition, decreased air-like density areas indicate improved intraoperative management of the operative field, including more effective evacuation and rational use of adjunctive materials. These imaging-based indicators provide objective evidence of technical advancement beyond conventional metrics.

### 4.4 Complications and clinical safety

Optimization along the learning curve was associated with a reduction in overall postoperative complications. Although differences in individual complications were not statistically significant, a consistent downward trend was observed, likely reflecting improvements in multiple aspects of surgical and perioperative management. These improvements may be attributed to more effective hemostasis, standardized blood pressure control, refined drainage management, and stricter adherence to sterile techniques. In addition, increased surgical precision and greater attention to peri-hematoma structures may have contributed to the reduction in procedure-related complications.

### 4.5 Influence of case complexity and surgical decision-making

An important consideration in surgical learning curve analysis is the potential evolution of patient complexity and operative indication selection throughout the learning curve As surgeons accumulate neuroendoscopic experience, increased technical confidence may gradually influence case selection, perioperative decision-making, and willingness to manage anatomically complex hematomas or patients with poorer neurological status.

In the present study, major baseline clinical characteristics, including hematoma volume, preoperative Glasgow Coma Scale score, ventricular involvement, and surgical timing, remained generally comparable across different learning phases without statistically significant differences in the principal severity indicators. These findings suggest that the observed improvements in operative efficiency and hematoma evacuation performance were more likely associated with progressive technical maturation, refinement of endoscopic manipulation, and optimization of perioperative workflow rather than major differences in patient selection.

Nevertheless, because of the retrospective single-center design, the possibility that increasing surgical experience subtly influenced operative indication judgment and perioperative management strategies cannot be completely excluded. Future prospective multicenter studies incorporating standardized severity stratification systems and longitudinal neurological outcome evaluation are warranted to further clarify the independent contribution of technical proficiency acquisition to surgical outcomes.

### 4.6 Clinical implications

The findings of this study have direct implications for surgical training. Approximately 25–30 cases appear necessary to achieve technical proficiency, consistent with the identified CUSUM inflection point. A staged training model is therefore recommended, progressing from basic skill acquisition to complex case management and individualized strategy development. Particular attention should be paid to the transitional phase, during which an overemphasis on evacuation rate may increase the risk of complications. Structured supervision and intraoperative decision support are essential during this period.

### 4.7 Limitations

This study has several limitations. First, this was a retrospective single-center observational study, which may inevitably introduce selection bias and limit the generalizability of the findings. Second, all procedures were performed by a single experienced neurosurgeon, and no direct comparison with surgeons from different institutions or with varying neuroendoscopic experience levels was available. Therefore, the present findings primarily reflect the learning trajectory and technical maturation process of an individual surgeon within a specific institutional setting.

Third, although baseline clinical characteristics remained generally comparable across different learning phases, the possibility that increasing surgical experience gradually influenced operative indication selection, perioperative decision-making, and management strategies cannot be completely excluded. As surgical proficiency increased, subtle evolution in case selection and confidence in managing anatomically complex hematomas or patients with poorer neurological status may have occurred during the learning curve.

Fourth, standardized long-term neurological outcome assessments, including modified Rankin Scale (mRS) and Glasgow Outcome Scale (GOS) evaluations at 3- or 6-month follow-up, were not consistently available because of the retrospective design and incomplete longitudinal follow-up during earlier surgical phases. Consequently, the present study primarily focused on technical proficiency acquisition, operative efficiency, hematoma evacuation performance, and perioperative safety rather than comprehensive long-term patient-centered neurological outcomes.

Finally, although the present study demonstrated progressive improvement in neuroendoscopic operative performance throughout the learning curve, the independent relationship between technical maturation and long-term functional neurological recovery requires further validation in prospective multicenter studies with standardized severity stratification systems and longitudinal outcome assessment protocols.

## 5 Conclusion

Neuroendoscopic evacuation of basal ganglia hemorrhage demonstrates a progressive learning curve characterized by improvements in operative efficiency, hematoma evacuation capability, and perioperative procedural stabilization. Increasing surgical experience was associated with progressive refinement of endoscopic manipulation, intraoperative coordination, and perioperative management strategies. Although further prospective multicenter validation incorporating standardized long-term neurological outcome assessment is warranted, the present findings provide valuable insight into the technical maturation process of neuroendoscopic hematoma evacuation and may contribute to optimization of surgical training and standardization of minimally invasive intracerebral hemorrhage management.

## References

[1] Xu M Tang C Shen Y Zhang Y Bao L. Analysis of the burden of intracerebral hemorrhage in the Asian population aged 45 and older and ARIMA model prediction trends: a systematic study based on the GBD 2021. Front Neurol. (2025) 16:1526524. 10.3389/fneur.2025.1526524 40027166 PMC11869383

[2] Wang JS Huang XF Yang WJ Chang SF. Clinical study of transfrontal keyhole approach with neuroendoscopic hematoma evacuation for intracerebral hemorrhage in the basal ganglia region. Chin J Minim Invasive Neurosurg. (2024) 28(12):728–31. 10.3969/j.issn.1005-6483.2024.04.014

[3] Kwon YH Jang SH. Changes in subcortical white matter in the unaffected hemisphere following unilateral spontaneous intracerebral hemorrhage: a tract - based spatial statistics study. J Integr Neurosci. (2022) 21(2):63. 10.31083/j.jin2102063 35364651

[4] Xiong JY Zhang HM. Effect and prognosis observation of preoperative DTI - assisted neuroendoscopic hematoma evacuation in the treatment of elderly patients with intracerebral hemorrhage. Hebei Medicine. (2025) 31(2):301–5.

[5] Huo G Lan Y Feng Y Gao X Chen C. The efficacy for hypertensive intracerebral hemorrhage between neuroendoscopic surgery and conservative treatment: a retrospective obsesrvational study. Neurologist. (2025) 30(2):109–15. 10.1097/NRL.0000000000000597 39575625

[6] Ren W Wang W Wang L Liu X Zhao Y. Efficacy and safety of minimally invasive neuroendoscopic surgery in the therapy of supratentorial hypertensive intracerebral hemorrhage: a meta - analysis. J Craniofac Surg. (2024) 35(8):2275–81. 10.1097/SCS.0000000000010529 39171781

[7] Wei HY Cai Y Wang C Li ZY Song P Zhou L. Research progress on surgical treatment of spontaneous supratentorial intracerebral hemorrhage. Stroke and Nervous Diseases. (2023) 30(4):418–22. 10.1136/svn-2024-003942

[8] Lobo K Santos C Campos P Oliveira L da Silva VEB. Neuroendoscopic surgery versus craniotomy for basal ganglia hemorrhage: a systematic review and meta - analysis of randomized controlled trials. Neurosurg Rev. (2025) 48(1):50. 10.1007/s10143-025-03213-w 39809946

[9] Lv K Wang Y Chao H Cao S Cao W. Comparison of the efficacy of subosseous window neuro - endoscopy and minimally invasive craniotomy in the treatment of basal ganglia hypertensive intracerebral hemorrhage. J Craniofac Surg. (2023) 34(8):e724–8. 10.1097/SCS.0000000000009461 37271862 PMC10597438

[10] Xu X Wei D Wang XF Chen G. Clinical efficacy of transfrontal keyhole approach with neuroendoscopic evacuation for hypertensive intracerebral hemorrhage in the basal ganglia region. J Mol Diagn. (2024) 16(12):2223–7. 10.3969/j.issn.1674-6929.2024.12.004

[11] Zhang Z Li Z. Application of neuroendoscopic surgery in treatment of hypertensive basal ganglia hemorrhage. Revista de Neurología. (2022) 75(5):109–16. 10.33588/rn.7505.2021445 35880964 PMC10280746

[12] Hou S. Clinical Efficacy Analysis of Gelatin Sponge Packing Technique in the Treatment of Massive Hypertensive Intracerebral Hematoma. Hefei: Anhui Medical University (2023).

[13] Guo X Liu MS Nan CR Shi YP Dong C Zong HQ. Evaluation of hemostatic performance of absorbable cellulose - based hemostatic materials with different textures and morphologies. Chinese Medical Equipment Journal. (2023) 38(2):126–30. 10.3969/j.issn.1674-1633.2023.02.023

[14] Gazzeri R Neroni M Pischedda M Martin M Galarza M Campagna D. A clinico-pathologic study of oxidized cellulose as topical hemostatic agent in neurosurgery. Surg Technol Int. (2015) 26:376–81. 26055035

[15] Wan Y Xie Q Hua Y Xi G Keep RF Pandey A. Comparing white and gray matter responses to lobar intracerebral hemorrhage in piglets and the effects of deferoxamine. Exp Neurol. (2025) 383:115041. 10.1016/j.expneurol.2024.115041 39489370

[16] Wang XG Lu YB Li X. Research progress on molecular mechanisms and treatment of white matter injury after intracerebral hemorrhage. Acta Physiologica Sinica. (2024) 76(1):59–76. 10.13294/j.aps.2024.0002 38444132

